# Effects of Population Dynamics on Establishment of a Restriction-Modification System in a Bacterial Host

**DOI:** 10.3390/molecules24010198

**Published:** 2019-01-07

**Authors:** Stefan Graovac, Andjela Rodic, Magdalena Djordjevic, Konstantin Severinov, Marko Djordjevic

**Affiliations:** 1 Faculty of Biology, University of Belgrade, 11000 Belgrade, Serbia; stefan.graovac@bio.bg.ac.rs (S.G.); andjela.rodic@bio.bg.ac.rs (A.R.); 2 Multidisciplinary PhD program in Biophysics, University of Belgrade, 11000 Belgrade, Serbia; 3 Institute of Physics Belgrade, University of Belgrade, 11080 Belgrade, Serbia; magda@ipb.ac.rs; 4 Waksman Institute of Microbiology, Rutgers University, Piscataway, NJ 08854, USA; severik@waksman.rutgers.edu; 5 Center for Life Sciences, Skolkovo Institute of Science and Technology, Skolkovo 143026, Russia

**Keywords:** restriction-modification systems, bacterial population dynamics, gene expression control, statistical thermodynamics, transcription regulation

## Abstract

In vivo dynamics of protein levels in bacterial cells depend on both intracellular regulation and relevant population dynamics. Such population dynamics effects, e.g., interplay between cell and plasmid division rates, are, however, often neglected in modeling gene expression regulation. Including them in a model introduces additional parameters shared by the dynamical equations, which can significantly increase dimensionality of the parameter inference. We here analyse the importance of these effects, on a case of bacterial restriction-modification (R-M) system. We redevelop our earlier minimal model of this system gene expression regulation, based on a thermodynamic and dynamic system modeling framework, to include the population dynamics effects. To resolve the problem of effective coupling of the dynamical equations, we propose a “mean-field-like” procedure, which allows determining only part of the parameters at a time, by separately fitting them to expression dynamics data of individual molecular species. We show that including the interplay between kinetics of cell division and plasmid replication is necessary to explain the experimental measurements. Moreover, neglecting population dynamics effects can lead to falsely identifying non-existent regulatory mechanisms. Our results call for advanced methods to reverse-engineer intracellular regulation from dynamical data, which would also take into account the population dynamics effects.

## 1. Introduction

Technological developments in the past few decades enabled experimental in vivo measurements of protein levels in single cells with high temporal resolution, thus providing a good basis for studying gene expression control by mathematical modeling [1,2]. The ultimate goal of these studies is to provide accurate predictions of gene expression dynamics in a cell, which is of particular importance for the emerging synthetic biology field. In particular, advances in fluorescence microscopy and microfluidics [1,3,4,5] allowed measurements of protein expression levels in a clonal culture descending from the same (single) cell, which abolishes the need to synchronize the cell population. First such measurements for bacterial restriction-modification (R-M) systems have recently become available [6].

R-M systems have two main components: restriction endonuclease (R) recognizes specific DNA sequences and cuts them, while methyltransferase (M) methylates the same sequences and thereby protects them from being cut [7,8]. R-M systems carried on plasmids can spread to new (naïve) bacterial hosts by horizontal gene transfer. Consequently, the synthesis of R and M has to be tightly controlled, to ensure safe and efficient R-M establishment in a naïve bacterial host. Specifically, R has to be synthesized with a delay with respect to M, so that the host genome is protected by M, before R activity appears [7,9]. Furthermore, since the same R-M system can be established in different bacterial species, system regulation must be at least partially independent from the host transcription factors, i.e., encoded by the system itself. This property to a certain extent simplifies identifying all system regulation components and correctly describing regulatory mechanisms.

We previously showed that biophysical and dynamical system modeling can reasonably explain available experimental data on R-M transcription control [10,11,12]. However, in vivo protein expression dynamics reflect not only transcription regulation, but also the (often neglected) effects of global physiological factors, which can change in the culture [13,14,15,16]. It was early pointed out that to understand function of cellular networks, they should be considered in their natural environment [17]. The global physiological factors can change significantly in the environment, which impacts the growth of the population of cells. Dilution of molecule concentrations upon cell division, and the changes in gene copy number, which are especially pronounced when the gene of interest is carried on a plasmid, are prominent growth rate dependent factors [18]. These effects can significantly modulate gene expression dynamics. For example, it was previously shown in the case of the celebrated *lac* operon that population dynamics effects have to be included in modeling of its gene expression to explain the measured data [19]. For a review of interdependence between cell growth and gene expression, studied in other systems, see [18] and references therein.

Therefore, the obtained time profile of protein expression is in reality a result of coupled specific regulatory, and global physiological factors that modulate the relevant population dynamics (dynamics of cell and plasmid division). The Esp1396I R-M system which will be analysed here, provides an interesting example of an unexpected protein expression time profile [6]. In this experiment, fluorescently labeled M and R amounts were tracked in time at the level of single cells in a microcolony originating from a single cell transformed with the system on a plasmid. The obtained time profile can only in part be explained by a model of known system regulation mechanisms [6]. The minimal theoretical model (which includes the experimentally established transcription regulation mechanisms, and dilution of molecular species in accordance with empirically observed bacterial population growth) explains the main qualitative characteristics of Esp1396I system expression dynamics: massive initial synthesis of methyltransferase and a delayed start of restriction endonuclease synthesis. However, while the model provides a good description of early-time dynamics, it does not agree with experiments at later times—see Figure 4C in [6]. For example, to explain the observed increase in intracellular amounts of M at later times, one would have to invoke an otherwise non-existing transcription activation mechanism. 

On the other hand, the bacterial cell division rate changes during the course of this particular experiment, so two growth regimes can be distinguished, in which the population grows exponentially, first with a higher and later, when the preferred nutrient is exhausted, with a lower cell division rate (see Figure 4A in [6]) [6,20]. In addition, the intracellular plasmid copy number dynamically increased during the experiment (unpublished observation). Consequently, there can be a highly non-trivial (and time dependent) interplay between cell and plasmid division rates, which might significantly affect the observed patterns of R and M dynamics. Such interplay (population dynamics effects) was neglected in our minimal model of Esp1396I expression dynamics, and is commonly neglected in modeling of bacterial intracellular dynamics. Therefore, before assuming that the unexplained properties of the measured dynamics are due to the action of some unknown regulatory mechanism(s) operating in the system, one should consider that they may arise from the already modelled regulation combined with the missing population dynamics effects. 

Consequently, a major goal of this research is to analyse the importance of population dynamics effects for intracellular dynamics. Understanding this is necessary to accurately predict gene expression dynamics in a cell, which is also crucial for a number of practical applications, ranging from synthetic biology [21,22] to bacterial antibiotic resistance [18]. This is here addressed on the example of Esp1396I R-M system gene expression control since: (*i*) state-of-the-art measurements of protein dynamics for this system are available [6]; (*ii*) our previous work showed that theoretical modeling can accurately explain R-M experimental measurements [6,10,11,12], and (*iii*) R-M systems are both important experimental model systems and sufficiently simple to be realistically theoretically modeled. In particular, the number of parameters is significantly lower to what is often encountered in models of gene expression networks/dynamics [23], and all the effects introduced in the model are supported by direct experimental observations [6]. 

## 2. Methods

### 2.1. Modeling cr Operon and m Gene Transcription Activities

**Figure 1:**
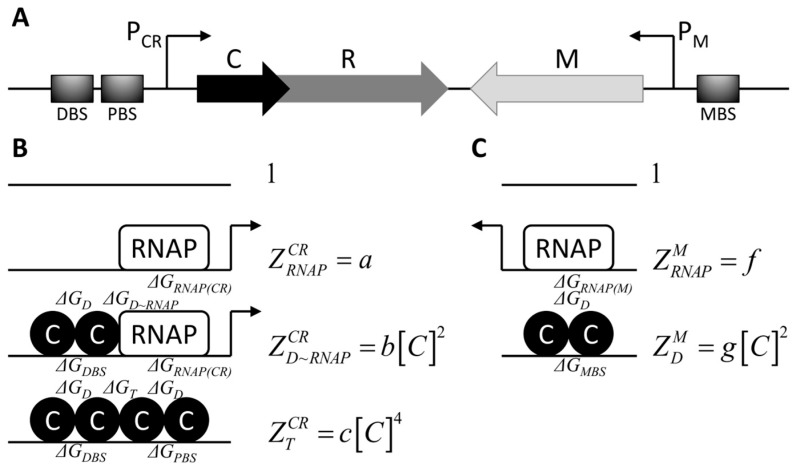
Modelling regulation of transcription in the R-M system Esp1396I. (**A**) Gene organization in the Esp1396I system: genes encoding control (C) protein and restriction endonuclease (R) are co-transcribed from the P_CR_ promoter, while the gene encoding methyltransferase (M) is transcribed from the P_M_ promoter. DBS (distal binding site), PBS (proximal binding site) and MBS (binding site in the P_M_) represent three binding sites for C protein, where PBS and MBS overlap with the corresponding core promoters (RNAP binding sites); (**B**) Allowed configurations of RNA polymerase (a rectangle denoted as “RNAP”) and C protein molecules (circles) on the P_CR_ and (**C**) the P_M_ promoters are schematically represented. Configurations in which the promoter is transcriptionally active are labelled with an arrow. The following free energies of protein-DNA and protein-protein interactions are indicated in the figure: Δ*G_RNAP(CR)_* and Δ*G_RNAP(M)_*—for binding of RNAP to P_CR_ and P_M_, respectively; Δ*G_DBS_*, Δ*G_PBS_* and Δ*G_MBS_*—for binding of a C dimer to the distal and the proximal binding sites, and to the binding site in the P_M_ region, respectively; Δ*G_D_*—for C protein dimerization; Δ*G_D~RNAP_*—for the interaction between the C dimer bound to the DBS and RNAP; Δ*G_T_*—for interaction between two bound C dimers. For each configuration, a statistical weight is assigned (on the right of the configuration), expressed in terms of C protein concentration ([C]) and constants a, b, c, f and g that are independent of C protein concentration.

The C protein is a transcription factor, encoded by Esp1396I [9,24], which regulates transcription of both the operon consisting of the *c* gene itself and the *r* gene (*cr* operon), and the methyltransferase (*m*) gene, as indicated in (Figure 1A). In particular, binding of the C protein dimer to a site partially overlapping with the strong P_M_ promoter of the *m* gene (denoted with MBS—for Methyltransferase Binding Site), represses *m* transcription (Figure 1C) [24]. 

The mechanism of *cr* transcription regulation is more complex (Figure 1B): at low C concentrations, C dimer is bound to the strong, distal binding site (DBS), and recruits RNA polymerase (RNAP) to the P_CR_ promoter thereby activating transcription of the *cr* operon (Figure 1B); at higher C concentrations, another C dimer is recruited to the adjacent weak, proximal binding site (PBS) forming a C tetramer on DNA, which represses transcription of *cr* [10,24].

Transcription activity of Esp1396I R-M system promoters P_CR_ and P_M_ depicted in Figure 1A is modelled using statistical thermodynamics, i.e., by representing the relevant molecular components in a cell as a system whose probability of different microstates is described by the Boltzmann distribution. Appropriate dimensionless statistical weights are assigned to each equilibrium configuration of RNA polymerase and C protein molecules at P_CR_ (Figure 1B) and P_M_ (Figure 1C), reflecting energetic and entropic costs of their establishment [25,26]. One should note that the same statistical weights can be obtained by applying the law of mass action to appropriate equilibrium binding reactions, where the equilibrium dissociation constants *K_d_*~exp(Δ*G*) (see [26] for a brief description of both approaches).

Statistical weights of the P_CR_ promoter configurations are given by the following Equations:

(1) $$Z_{RNAP}^{CR}=k\left[\mathrm{RNAP}\right]\exp\left(-\Delta G_{RNAP(CR)}\right)$$

(2) $$Z_{D\sim RNAP}^{CR}=k^{3}{\left[C\right]}^{2}\left[\mathrm{RNAP}\right]\exp\left(-\Delta G_{DBS}-\Delta G_{RNAP(CR)}-\Delta G_{D}-\Delta G_{D\sim RNAP}\right)$$

(3) $$Z_{T}^{CR}=k^{4}{\left[C\right]}^{4}\exp\left(-\Delta G_{DBS}-\Delta G_{PBS}-2\Delta G_{D}-\Delta G_{T}\right)$$

where statistical weights in Equations (1)–(3) correspond, respectively, to: (*i*) only RNA polymerase bound to the promoter, corresponding to basal transcription of the genes encoding C protein and restriction endonuclease (Equation (1)), (*ii*) RNA polymerase recruited to the promoter by the C dimer bound to the DBS (Equation (2)), and (*iii*) a second C dimer recruited to the PBS by the C dimer bound to the DBS, forming a C tetramer on DNA which represses transcription (Equation (3)). In the upper equations, *k* is a proportionality constant (with units one over concentration), concentrations of molecular species are labelled with square brackets, while protein-DNA and protein-protein interaction energies that enter Equations (1)–(3), (Δ*Gs* in the units of *k_B_T*) are denoted in Figure 1B and listed in the caption of the Figure. Constants in Equations (1)–(3) can be absorbed into few parameters (*a*, *b* and *c*) that do not depend on C concentration, which results in the expressions for statistical weights denoted next to the appropriate configurations in Figure 1B. 

The standard assumption in thermodynamic modeling is that transcriptional activity of the promoter is proportional to the equilibrium probability that RNA polymerase is bound to that promoter [27]. Accordingly, transcriptional activity of P_CR_ is given by the following equation (1 is the statistical weight corresponding to the empty promoter):

(4) $$\varphi_{r}=\alpha \frac{Z_{RNAP}^{CR}+Z_{D\sim RNAP}^{CR}}{1+Z_{RNAP}^{CR}+Z_{D\sim RNAP}^{CR}+Z_{T}^{CR}}$$

where *α* is a proportionality constant with units of transcript amount over time. Equation (4) can be rewritten introducing the derived statistical weights from Figure 1B, and defining the basal transcription activity of the P_CR_ in the absence of C protein, $\varphi_{r}^{basal}=\alpha \cdot a/(1+a)$:

(5) $$\varphi_{r}(C)=\varphi_{r}^{basal}\frac{1+\frac{b}{a}{\left[C\right]}^{2}}{1+\frac{b}{a+1}{\left[C\right]}^{2}+\frac{c}{a+1}{\left[C\right]}^{4}}.$$

Transcription activity of P_M_ promoter is modelled in the same manner (Figure 1C), assuming that it can be found: (*i*) empty, (*ii*) in a transcriptionally active state when it is occupied by RNA polymerase, or (*iii*) in a repressed state, when a C dimer is bound to the MBS. Statistical weights of the configurations described under (*ii*) and (*iii*) read:

(6) $$Z_{RNAP}^{M}=k\left[\mathrm{RNAP}\right]\exp\left(-\Delta G_{RNAP(M)}\right)$$

(7) $$Z_{D}^{M}=k^{2}{\left[C\right]}^{2}\exp\left(-\Delta G_{MBS}-\Delta G_{D}\right)$$

while the P_M_ transcription activity is given by the equation:

(8) $$\varphi_{m}=\beta \frac{Z_{RNAP}^{M}}{1+Z_{RNAP}^{M}+Z_{D}^{M}}$$

where *β* is a proportionality constant with units of transcript amount over time. As in the case of the P_CR_ activity modeling, constants in Equations (6) and (7) can be absorbed into few parameters (*f* and *g*) that do not depend on C concentration, which results in the expressions for statistical weights denoted next to the appropriate configurations in Figure 1C. Equation (8) becomes

(9) $$\varphi_{m}(C)=\varphi_{m}^{basal}\frac{1}{1+K_{i}^{2}{\left[C\right]}^{2}}$$

when $K_{i}^{2}=g/(f+1)$ and $\varphi_{m}^{basal}=\beta \cdot f/(f+1)$ are introduced. Note that *K_i_* corresponds to the equilibrium association constant for C dimer binding to DNA in the presence of a bound RNAP, which exerts an inhibiting effect on transcription.

### 2.2. Introducing the Interplay of Cell and Plasmid Division Rates

We here develop a model that includes an interplay of cell and plasmid division rates, so that full population dynamics effects due to (time dependent) division of both cells and plasmids are taken into account. Note that R-M systems typically produce thousands of RNAs and proteins of the two enzymes in the cell, so these systems can be reliably modelled deterministically. 

The number of R-M system encoding plasmids per cell (*n_p_*) is introduced as a time dependent variable, which increases due to plasmid replication, and decreases due to dilution after cell division. Specifically, the change of cell numbers (*n_cell_*), and the number of plasmids per cell (*n_p_*) with time is described by the following differential equations:

(10) $$\frac{dn_{cell}}{dt}=\lambda_{cell}\cdot n_{cell}$$

(11) $$\frac{dn_{p}}{dt}=\left(\lambda_{p}-\lambda_{cell}\right)\cdot n_{p}$$

where *λ_cell_* and *λ_p_* are division rates for cells and plasmids, respectively. Experimentally measured cell number dependence with time [6] indicates a relatively sharp transition from faster to slower cell division rate, happening at $t_{cell}^{trans}$ ~150 min. To obtain a curve that can satisfactory describe the measured data, the area of transition is interpolated using the hyperbolic tangent function. Note that this interpolation covers a relatively short time interval ~30 min, so it does not significantly affect the predictions, but is necessary to avoid discontinuity when solving differential equations—moreover, it naturally extrapolates between the two regimes of cell division. In particular, *λ_cell_*(*t*) is determined as follows:

(12) $$\begin{array}{l} w_{cell}=\frac{1}{2}+\frac{1}{2}\cdot \tanh\left(\frac{t-t_{cell}^{trans}}{s_{cell}}\right)\\ \lambda_{cell}(t)=w_{cell}\cdot \lambda_{cell2}+\left(1-w_{cell}\right)\cdot \lambda_{cell1} \end{array}$$

where *λ_cell_*_1_, *λ_cell_*_2_ are constant parameters denoting the cell division rates in the first and the second time interval, while *s_cell_* is a fixed parameter defining smoothness of the interpolation (taken as 120 min). Note that Equation (12) is just an empirical fit (basically continual interpolation) to the experimentally measured data, which is an input to the model of the gene expression dynamics, rather than a model prediction.

The plasmid division rate *λ_p_*(*t*) is introduced in an analogous manner:

(13) $$\begin{array}{l} w_{p}=\frac{1}{2}+\frac{1}{2}\cdot \tanh\left(\frac{t-t_{p}^{trans}}{s_{p}}\right)\\ \lambda_{p}(t)=w_{p}\cdot \lambda_{p2}+\left(1-w_{p}\right)\cdot \lambda_{p1} \end{array}$$

with equivalent parameter labelling as in Equation (12). Finally, in the full dynamical model, the number of plasmids is obtained by solving Equations (11) and (13), and then multiplied by promoter transcription activities to obtain the generation rates of the corresponding RNA species. The full model is described with the following set of differential equations:

(14) $$\frac{dr}{dt}=n_{p}\cdot \varphi_{r}(C)-\lambda_{r}\cdot r$$

(15) $$\frac{dm}{dt}=n_{p}\cdot \varphi_{m}(C)-\lambda_{m}\cdot m$$

(16) $$\frac{dC}{dt}=k_{C}\cdot r-\lambda_{C}\cdot C$$

(17) $$\frac{dR}{dt}=k_{R}\cdot r-\lambda_{R}\cdot R$$

(18) $$\frac{dM}{dt}=k_{M}\cdot m-\lambda_{M}\cdot M.$$

Equations (14) and (15) describe how the amounts of transcripts of the *cr* operon and the *m* gene change with time, while Equations (16)–(18) describe the same for the amounts of proteins, namely, of C protein (C), restriction endonuclease (R) and methyltransferase (M). For parameter notation, see Table 1. Note that each transcript and protein decay rate (*λ*’s in the equations above) represents a sum of *λ_cell_* (Equation (12)) and the corresponding molecule degradation rate (which we mark by ~ signs, e.g., $\lambda_{r}=\tilde{\lambda_{r}}+\lambda_{cell}(t)$). Since we take protein decay rates of R and C to be equal, Equation (17) can be excluded from solving, i.e., replaced by an algebraic relation $R=C\cdot k_{R}/k_{C}$ (note that C and R in Equations (16) and (17) can be rescaled with *k_C_* and *k_R_*, respectively, leading to the same equation). 

### 2.3. Numerically Solving and Fitting the Model Equations

If the population dynamics effects, in particular changes of per cell plasmid copy number throughout the experiment, would be neglected [6], the equations for R (and consequently also C, see above) dynamics could be solved separately from the equation for M dynamics. Once C dynamics are solved, they could be used to determine the M dynamics (since the *m* gene is regulated by C, see Equations (15) and (18)), as was done with the minimal model provided in [6]. However, the introduction of plasmid dynamics in the model leads to nontrivial coupling of R (Equations (14), (16) and (17)) and M dynamics (Equations (15) and (18)) through the time-dependent term *n_p_* that enters both Equations (14) and (15). Consequently, these sets of equations have to be solved simultaneously, and their parameters can no longer be separately fitted to the experimental data for R and M dynamics. This then significantly increases dimensionality of the parameter inference problem, i.e., the joint fit leads to inferring parameters in a 17-dimensional space, which is computationally complicated, since a very large number of parameter combinations have to be explored to find the best fit of the model to experimental data.

**Figure 2:**
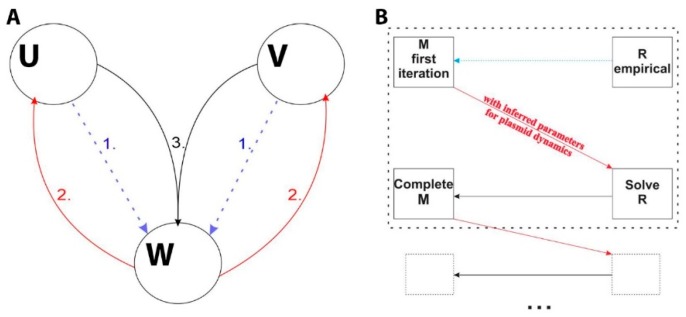
Schematic representation of “mean field-like” procedure for reducing dimensionality of the parameter inference problem for the model with population dynamics effects included. (**A**) Generalized procedure. Circles represent sets of ODEs describing dynamics of particular species (here labeled by U, V and W). The blue dashed arrows denote that in the first step, dynamics of species U and V are empirically approximated and used to determine the parameters of W dynamics by solving only W related ODEs. In the next fitting step (indicated by the red solid arrows and the number 2), the shared parameters (those describing population dynamics) inferred by fitting in the previous step are used in fitting the solution of U and V related ODEs and thereby inferring their corresponding parameters. In the step 3, the fitted (and not the empirically approximated) solution of the U and V related ODEs is used in inferring the parameters of W dynamics. The steps 2 and 3 repeat iteratively until the best fit of the whole model to the data is obtained; (**B**) Applying the procedure to the case of R-M dynamics: boxes represent consecutive fitting steps, and arrows indicate the order of solving the sets of equations corresponding to R and M dynamics. Dashed arrows and squares indicate that the steps in the upper box can be iteratively repeated until convergence is reached. The arrow style and labeling is equivalent as in part A of the Figure.

To resolve this problem, which would become even more prominent with a larger number of species in the model, we here propose an iterative, “mean field-like” approach to effectively decouple such equations. The main idea is schematically depicted in Figure 2A, and in essence corresponds to solving for dynamics of only one molecular species (W in Figure 2A), in the “field” obtained by empirically approximating the dynamics of the other species (U and V and arrow 1 in Figure 2A). This then allows inferring the population dynamics parameters (which couple the species dynamics), by fitting the model only to experimental data for W dynamics. Once these population dynamics parameters are fixed, one can then return (arrow 2 in Figure 2A) to other species, and separately solve for their dynamics, by fits to corresponding experimental data. With solved dynamics for U and V, one then goes back (arrow 3) and solves W dynamics: (*i*) with either fixed (previously inferred) parameters for population dynamics, in the case that the procedure converges, i.e., leads to a satisfactory fit to experimental data or (*ii*) by refitting (inferring again) the population dynamics parameters, if the convergence has not been achieved—in this case, the whole procedure is further iteratively repeated until convergence. 

The application of this procedure to R-M dynamics is depicted in Figure 2B. Note that the time dependence of R directly determines the time dependence of C, as they are proportional to each other (see the previous subsection). The procedure is started by empirically fitting the time dependence of R, as it is simpler compared to M. In fact, it can be well approximated by quadratic dependence on time. On the other hand, a more complex time dependence of M data is more suitable (i.e., likely more sensitive) to inferring the population dynamics parameters, in addition to fit being of smaller dimensionality compared to R. Once C dynamics is empirically fitted, the Equations (15) and (18) for M dynamics are solved and the solution is fitted to corresponding experimental data, from which the plasmid dynamics parameters are inferred. Then, one can use these parameters to solve for R dynamics, and thereby determine the rest of the parameters in R dynamics. One then goes back to M dynamics and solves it by using the newly inferred C(*t*). If the inferred (fixed) parameters of M dynamics lead to a satisfactory agreement with experimental data (converge) the procedure is stopped. If not, the plasmid dynamics parameters are again estimated (with C(*t*) from the previous step). Note that after each full cycle, the solutions for both M(*t*) and R(*t*) (i.e., C(*t*)) exactly solve the full system of the dynamical equations, though the parameters inferred after each cycle may not provide an optimal fit to data—thus, the fit is, if needed, improved through iterative cycles. 

To implement the above procedure, the system of differential Equations (11), (14)–(16) and (18) that represents the full model of Esp1396I R-M system is solved numerically using the Runge-Kutta method [28]. The initial conditions are set to zero for all species except for plasmids, for which one plasmid per cell at the beginning of the experiment was set. The system parameters are varied in the ranges that correspond to biochemically realistic values [25]. The set of parameters that best fits experimental data was determined by minimizing R^2^ (the sum of the squared errors). Note that this numerical procedure is quite more complicated than standard fits to experimental data, as in our case there is no closed form expression that can be fitted to the data points. That is, the closed form expressions for transcription activities (Equations (1)–(9)) serve as an input for non-linear differential equations (Equations (14)–(18)), which cannot be integrated in a closed form expression, but have to be solved numerically, and these solutions then compared with experimental data.

Тo quantify the model comparison with experimental data, adjusted R^2^ was calculated for each fit. To quantitatively compare different fits (e.g., for “complex” and “simple” models), F values and corresponding P values used [29]:

(19) $$F=\left(\frac{\chi_{1}^{2}-\chi_{2}^{2}}{p_{2}-p_{1}}\right)/\left(\frac{\chi_{2}^{2}}{n-p_{2}}\right)$$

where $\chi_{1}^{2}$ and $\chi_{2}^{2}$ are the sum of residuals squared for the first (“simple”), and the second (“complex”) model; *p*_1_ and *p*_2_ are the corresponding numbers of parameters, while *n* is the number of data points. From this, we can estimate the corresponding P-values from cumulative distribution function of F statistics.

## 3. Results and Discussion

### 3.1. Including the Population Dynamics Effects

The minimal model from [6] was redeveloped to include the effects of simultaneous cell and plasmid division. However, the associated coupling of R and M dynamics brought a technical challenge. In particular, since the system genes are located together on a plasmid (see Equations (14)–(18)), the corresponding equations for R and M can no longer be solved (and fitted to experimental data) separately. This in turn resulted in a significant increase in the dimensionality of the parameter inference problem (to 17). This technical difficulty would become even more pronounced in a model with a larger number of species, where their dynamics would become coupled due to introducing population dynamics effects. To solve this problem, we here proposed a “mean field-like” iterative approach in which the dynamics of two enzymes is effectively decoupled by first empirically approximating dynamics of species 1 and using it to solve for the parameters related to the dynamics of species 2, including those describing the population effects. 

Next, the parameters inferred for species 2 are used to solve the equations for species 1 and estimate the appropriate parameters of its regulation. The new species 1 parameters are then used to solve again for the species 2 parameters, and the process is repeated iteratively until the best fit to both species dynamics data is obtained. Note that this “mean field-like” procedure can be generalized to multiple molecular species, as described in Methods (see Figure 1A). In addition to a much simpler parameter inference, note that the decreased dimensionality of the parameter space, also leads to generically smaller chance to overfit the data (due to a smaller number of parameters involved in the fit).

**Figure 3:**
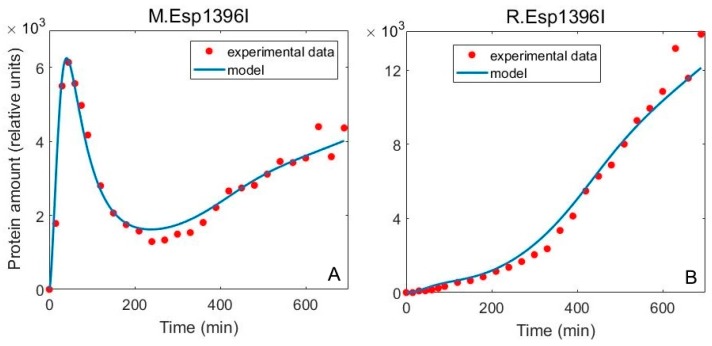
Comparison of the model with included population dynamics with experimental data. Comparison of predictions for Esp1396I R-M system with experimentally measured time-dependence of protein amounts in single cells. The model (blue curve) is fitted to experimental data (red dots) for (**A**) methyltransferase (adjusted R^2^ = 0.97) and (**B**) restriction endonuclease dynamics (adjusted R^2^ = 0.98).

Applying this procedure to Esp1396I system data leads to a much better agreement of the model with experiments (Figure 3A), compared to the fit of the minimal model (Figure 4C in [6]). In particular, the adjusted R^2^ for the two fits in Figures 3A and 4C from [6], are respectively, 0.97 and 0.25, where F value (160) for the fit comparison is statistically highly significant (P~10^−15^). The increase of M later in the experiment, which is now accounted for, is a consequence of slower cell division compared to plasmid replication later in the experiment and not due to overfitting (Figure 3A). These results also demonstrate the suitability of “the mean field–like” procedure for reducing the dimensionality of the parameter inference problem. The inferred parameter values (see Table 1) are also in general agreement with experimental observations: high stability of R-M proteins predicted by the model is in a direct agreement with experimental observations [6]; the inferred decay rates are also in accordance with the standard expectation that mRNAs are more rapidly degraded than proteins [30]; the obtained three times lower translation rate of *cr* compared to *m* is consistent with providing a robust delay of R with respect to M, and such mechanism was also found in AhdI R-M system [10]. The errors for the parameter fit values could be estimated through either Monte Carlo or bootstrapping procedure. While this is in principle straightforward, it is in practice highly computationally intensive, as the system has to be simulated for all the parameter combinations and for all of the many generated synthetic datasets [31]. 

### 3.2. Including Population Dynamics Improves Agreement with the Expression Measurements

**Figure 4:**
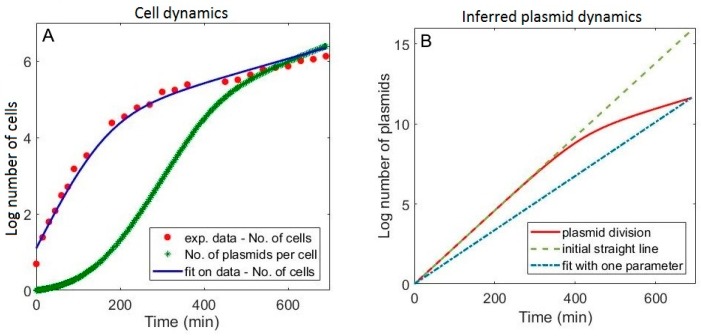
Population (cell and plasmid) dynamics. (**A**) Experimentally measured log number of cells vs. time (red dots) fitted by equation (12) (blue curve). The average number of plasmids per cell in time (green crosses), is shown on the same graph to allow comparison; (**B**) Solid red curve corresponds to the plasmid dynamics from Equation (13), described by three parameters (in analogy to the experimentally observed cell dynamics). Green dashed line represents the plasmid dynamics generated by the constant plasmid division rate, which corresponds to extrapolating the early part of the red curve to the end of the experiment. Blue dash-dotted line also corresponds to the assumption of constant plasmid division rate, where the division rate is taken as a free parameter obtained by fitting the model to R and M dynamics data.

We next address if the number of parameters in the model is minimal, i.e., if experimental data could be explained with a less complex model. Namely, plasmid dynamics were modelled with three parameters representing the plasmid division rate in the first time interval (*λ_p_*_1_)), the plasmid division rate in the second time interval (*λ_p_*_2_), and the time of transition between the two intervals ($t_{p}^{trans}$). Such dependence is analogous to experimentally measured cell division rate, where two intervals with almost constant division rates (transition at ~150 min; see Figure 4A) are separated by a transition period. By comparing Figure 4A,B, one can see that plasmid dynamics providing the best fit of the model to the data comprise a late (at ~400 min; see Figure 4B) transition between the two intervals with constant plasmid division rates. This inferred transition of the plasmid division rate from higher to lower value is consistent with general notion of positive correlation between the cell division rate and gene copy number increase [18], and with the fact that the cell division rate becomes slower later in the experiment (see Figure 4A).

We next test if the data can be explained by a simpler model of plasmid dynamics, with a smaller number of parameters. To that end, we try to describe plasmid division with only one parameter (instead of three), specifically, with a constant plasmid division rate throughout the course of the experiment. In the three parameter model, the plasmid division dynamics are dominated by the first division rate (see the full curve in Figure 4B). Accordingly, we test if the late decrease in the plasmid division rate is dispensable in terms of explaining the data, by assuming that the obtained value of the first plasmid division rate (describing the early plasmid dynamics) remains constant during the whole course of the experiment (see the dashed line in Figure 4B). 

This one-parameter assumption clearly leads to a poorer agreement of the model with the data points in the late phase of the experiment (Figure S1A,B in the Supplementary Material), emphasizing that a finer description of plasmid division dynamics is important for explaining the experiment. Further, we model plasmid division dynamics with one parameter which now becomes free, and fit the whole gene expression model to the experimental data. However, such a model also cannot provide a good fit to the data (Figure S1C,D), where the disagreement is particularly pronounced for M expression dynamics (Figure S1C)—note that F (and corresponding P) values for comparison with fits in Figure 3) is provided in caption of Figure S1. Overall, neither of alternative possibilities can provide good agreement of the model with the data, implying that the original (three-parameter) model is indeed necessary (minimal) to explain the data.

**Figure 5:**
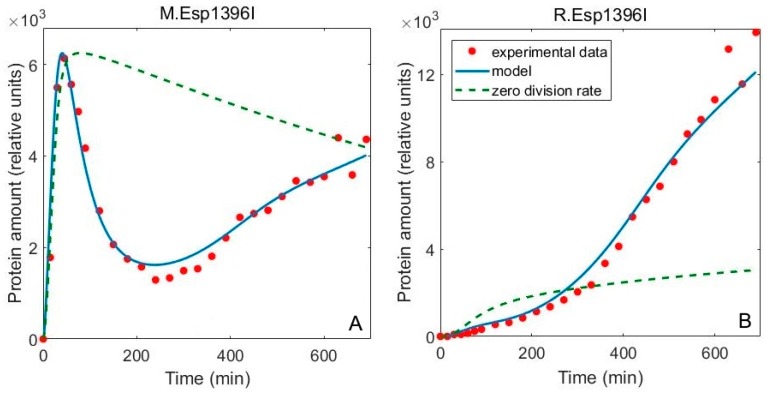
Excluding population dynamics from the full model. The blue solid line represents the full model fitted to experimental data (red dots) for (**A**) methyltransferase and (**B**) restriction endonuclease, while the green dashed line is obtained by excluding the population dynamics from the model and running the simulation with unchanged values for the rest of the parameters. To better compare the shape of the curves, the model without the population dynamics is rescaled, so that the maximal value reached by M is the same as in the full model.

To further test to what extent the included population dynamics are necessary to explain the data, we assess their contribution in establishing the final protein expression pattern. To this end, cell and plasmid division rates were set to zero in the full model, so that the model only describes specific gene regulation in the system. Simulation of the model in this case results in notably qualitatively changed protein dynamics for both M and R, which poorly fit the data (Figure 5). As M in the model is very stable, its amount decreases very slowly, governed by transcription repression by the C protein (Figure 5A). As expected, an increase in the amount of M later in the experiment cannot be recovered by the model which includes transcription regulation alone. 

If one would attempt to explain the increase in the amount of M later in the experiment through transcription regulation alone, a non-existent activation of the P_M_ promoter at higher C protein concentrations would have to be invoked. Furthermore, one may misinterpret the observed rapid M decrease after the peak, as an indication of highly cooperative repression of P_M_, since a trademark of high cooperativity is a sharp transition of the system from the ON to the OFF (or vice-verse) state [30]. However, this dynamical property is clearly a result of the population effects, since the Figure 5A suggests that without the population dynamics the peak in M dynamics would be much broader. The same (even more drastic) result is obtained if the model without population dynamics would be refitted to the experimental data (see Figure S2B).

With regards to the R dynamics, if the population dynamics effects are neglected, R slowly increases to some saturation value (Figure 5B), while the shape of the curve is concave instead of the convex form observed in the experiment. Namely, the rapid increase of R late in the experiment is due to two population dynamics effects, which overcome repression by C exhibited at later times. In particular, (i) the rate of cell division slows down at later times (Figure 4B), lowering the effective R decay rate, and (ii) the number of plasmids per cell keeps increasing, leading to increased generation of R transcripts. Both of these effects clearly promote the increase of R at later times counteracting the repression by C, which provides an explanation for the experimentally observed time dependence. 

To further check if the model describing only internal system regulation can explain the observed dynamics, it was re-fitted to the experimental data of measured protein expression (Figure S2 in Supplementary material). It can be analytically shown that the observed R dynamics can be explained by such a model only under conditions that transcripts and proteins are very stable and that there is no regulation of the P_CR_ promoter by C protein (Figure S2A). Clearly, this good fit does not provide a correct picture of experimentally inferred regulation in the system. In addition, this model gives a very poor fit to the experimental data for M expression (Figure S2B)—for M the adjusted R^2^ corresponding to 0.22, with F value for the fit comparison with Figure 3A of P~10^−13^. Together, these results strongly suggest that the observed Esp1396I system expression dynamics cannot be explained solely by the system specific regulation, and provide an additional argument that the data do not become over fitted by including the population dynamics effects.

Overall, it can be argued that population dynamics, while often neglected, significantly influences the expression dynamics of the system, and is crucial for understanding the system dynamics. Ignoring such dynamics can lead to identification of non-existent regulatory mechanisms of gene transcription in an effort to intuitively interpret experimental data.

### 3.3. Falsely Identifying Regulation when Plasmid Dynamics is not Included

We next analyse how neglecting only the plasmid division dynamics affects the observed kinetics of protein synthesis. Instead of focusing on quantitative comparison of the model predictions with the data, we will focus on how neglecting the plasmid dynamics can lead to false identification of regulatory mechanisms that in reality do not exist.

**Figure 6:**
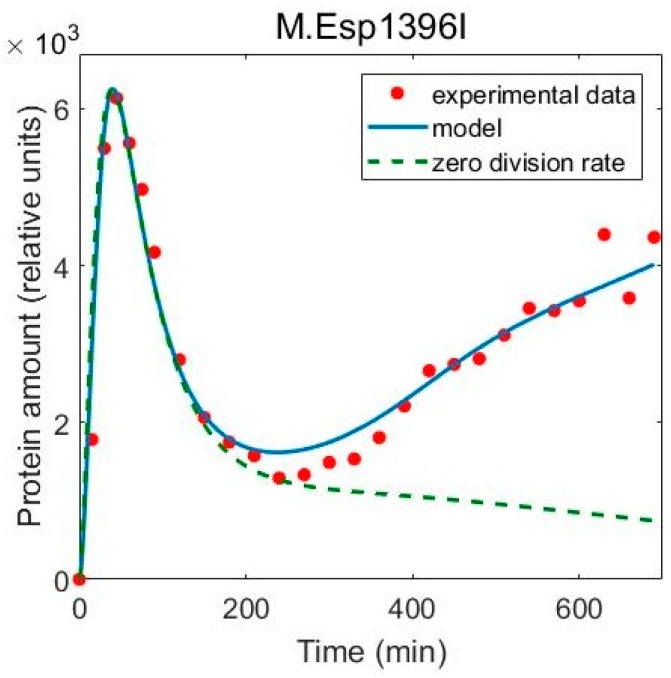
Effect of plasmid dynamics on intracellular M amounts. Experimentally measured intracellular M.Esp1396I amounts at different time points after transformation are denoted by red dots. The solid blue curve is the full model prediction, while the dashed green curve is obtained by setting the plasmid division rate in the full model to zero.

We start by observing how modeling agrees with M dynamics data when plasmid dynamics are included (full line), and when they are excluded from the model (dashed line), which is shown in Figure 6. After ~200 min the M amount in the model without plasmid dynamics slowly decreases over time, while the experimental data show that the amount of this enzyme starts to increase.

Consequently, one can speculate that a single, major effect behind the increase of M in the second interval of the experiment is plasmid dynamics, i.e., increase in the plasmid numbers late in the experiment. Neglecting plasmid dynamics in modeling can thus lead to (qualitative) misinterpretation of experimental results. For example, an increase in the amount of M later in the experiment can be interpreted as a consequence of non-existent P_M_ activation at higher concentrations of C protein, while in reality there is only repression [24]. 

**Figure 7:**
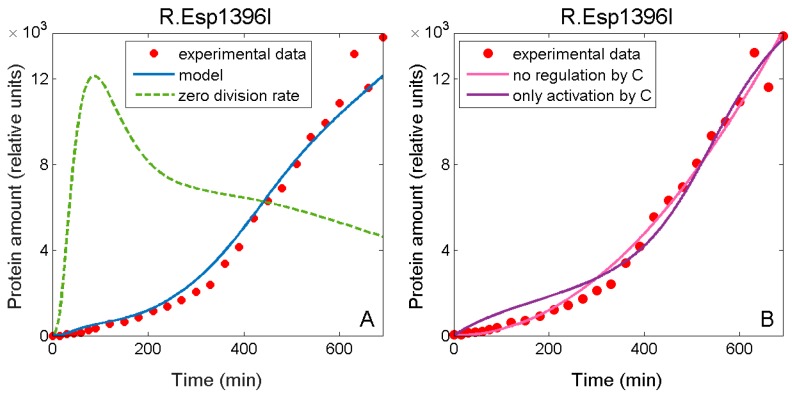
Effects of neglecting plasmid dynamics on intracellular R amounts. (**A**) Fit of the full model, taking into account plasmid division dynamics, to R data (red dots) is given by a solid blue curve, while the green dashed curve is obtained by setting the plasmid division rate to zero—the green curve is rescaled so that it can be compared with the blue one; (**B**) Two attempts to fit the R.Esp1396I data relying on intuitive expectations based on the form of the experimentally measured trajectory (all population dynamics effects are here neglected): (i) the model implied by the analytical derivation, which completely ignores P_CR_ promoter regulation by C protein, generates the solid pink curve, (ii) the best fit of the model assuming standard rates of protein and transcript degradation and neglecting transcription repression by C protein is given by the solid purple curve.

Similar holds when explaining the properties of R dynamics (Figure 7A): excluding plasmid replication from the model describing R expression results in a visibly different dynamics curve from that observed experimentally. Notably, experimental data for R dynamics can be empirically modelled with a quadratic equation. This time dependence can be derived analytically in the absence of population dynamics effects, if proteins and transcripts are assumed entirely stable, and if there is no regulation by C protein (see the pink curve in Figure 7B). Since a specific regulatory mechanism exploiting C proteins was confirmed in a number of previous experiments on the Esp1396I system [24,32,33], such a derivation obviously leads to a wrong conclusion about system expression control. Furthermore, the measured R dynamics naively look as arising from only transcription activation by C protein operating in the P_CR_ promoter control (as R amounts monotonically increase with time), but from a quantitative viewpoint, modeling such a case provides a significantly worse fit to the data (purple curve in Figure 7B)—F value for the comparison with the fit in Figure 3A is 25 (P~10^−7^). Therefore, intuitive reasoning may be in disagreement with a true situation in a cell.

R dynamics are, therefore, a nice example for apparently simple (quadratic) time dependence generated by a complex interplay between very different opposing effects, in particular, those arising from intracellular regulation (repression at higher C concentrations) and population dynamics (the increase in plasmid numbers later in the experiment). This interplay could not be inferred intuitively, since an intuitive interpretation would suggest only activation of the *cr* operon by C protein. Moreover, it could not be inferred even through analytical derivations, since they imply a constitutive (non-regulated) gene expression, and stable RNA and protein amounts, which is very different from reality. Therefore, an accurate understanding of the system regulation and the resulting dynamics requires taking into account all relevant effects, and their careful computational modeling.

## 4. Conclusions

An earlier minimal model of the Esp1396I R-M system expression predicted that the methyltransferase amount changes oppositely in time to what was experimentally observed in microcolonies grown from single transformed cells. The disagreement could have been interpreted as a consequence of unknown regulatory mechanism(s) operating in the system, that were not accounted for in the model. However, from our analysis it follows that the reason behind this disagreement are the commonly neglected population dynamics effects—namely, kinetics of cell division and plasmid replication—which, when included, lead to a very good agreement with the data. Consequently, neglecting global physiological effects on gene expression can lead to falsely identified regulation by significantly impacting qualitative properties of intracellular protein expression dynamics.

From a computational perspective, we note that including population dynamics in the model, which is necessary to explain the experimental data, is a highly nontrivial task, as it inevitably increases dimensionality of the parameter inference. Still, we showed that this problem can be approached through a procedure in which the shared population dynamics parameters are initially estimated by considering dynamics of only one of the multiple species coupled by the regulation mechanisms, relying on approximated dynamics of the rest of the species. Such a “mean field-like” procedure can become even more necessary when considering larger gene regulatory networks, as dimensionality of the parameter inference problem would further increase from the one considered here. While the procedure is here introduced in the form that can be directly applied to any number of molecular species, it remains to be tested in practice when applied to more complex regulatory networks. 

As an outlook, we have seen here that population dynamics effects, which are modulated by changes in global physiological factors, can have a significant effect on gene expression dynamics. Consequently, expression patterns of molecular species within a cell can result from a complex interplay of intracellular regulation and population dynamics effects, as demonstrated here in the case of the Esp1396I system. This then calls for advanced methods to reverse-engineer intracellular regulation from dynamical data, which would take into account both intracellular regulation and effects due to changing external conditions [13,18,20,34]. One approach used to filter out the effects of the global physiological state of the cell, which directly impact population dynamics parameters, involves experimentally tracking expression from a constitutive and from a regulated promoter in parallel [16]. In addition, varying of gene copy number with time (e.g., due to kinetics of plasmid division) should be accounted for by both experiments and theoretical models. A particular challenge would be to reconstruct regulatory networks from sole protein dynamics data, that are becoming more and more available [35]. Development of advanced theoretical methods for such reconstruction, analogous to those for reverse engineering of gene networks from gene expression data, may thus become a necessity in the future.

## Figures and Tables

**Table 1:** Model parameters.

Notation	Value	Description
**Population Dynamics**		
$\lambda_{cell1}$	2.3 × 10^−2^	Cell division rate in the first time interval
$\lambda_{cell2}$	3.1 × 10^−3^	Cell division rate in the second time interval
$t_{cell}^{trans}$	1.5 × 10^2^	Time of transition between the cell division rates
$\lambda_{p1}$	2.3 × 10^−2^	Plasmid division rate in the first time interval
$\lambda_{p2}$	7.5 × 10^−3^	Plasmid division rate in the second time interval
$t_{p}^{trans}$	4.2 × 10^2^	Time of transition between the plasmid division rates
**Restriction endonuclease dynamics**		
$\varphi_{r}^{basal}$	5.3 × 10^−1^	Basal transcription activity of the P_CR_ promoter
a	2.7 × 10^−1^	Constants which absorb the relevant interaction energies and RNA polymerase concentration
b	4.3 × 10^−3^	
c	2.7 × 10^−9^	
$k_{C}$	5.1 × 10^−1^	Translation rate for control protein
$k_{R}$	$=k_{C}\cdot 2/3$	Translation rate for restriction endonuclease
$\lambda_{r}$	3.8 × 10^−2^	Decay rate for the operon transcript
$\lambda_{C}$	1.2 × 10^−6^	Decay rate for control protein
$\lambda_{R}$	$=\lambda_{C}$	Decay rate for restriction endonuclease
**Methyltransferase dynamics**		
$\varphi_{m}^{basal}$	4.6 × 10^1^	Basal transcription activity of the P_M_ promoter
$K_{i}$	3.0 × 10^−5^	Constants which absorb the relevant interaction energies and RNA polymerase concentration
$k_{M}$	1.5	Translation rate for methyltransferase
$\lambda_{m}$	2.0 × 10^−1^	Decay rate for the *m* gene transcript
$\lambda_{M}$	8.0 × 10^−4^	Decay rate for methyltransferase

Time is measured in minutes, while the rates are given in 1/min.

## Supplementary Materials

The following are available online: Figures S1 and S2.
