# Supplementary material for: Effects of Population Dynamics on Establishment of a Restriction-Modification System in a Bacterial Host
Source: Molecules. 2019 Jan 7;24(1):198. doi: 10.3390/molecules24010198 (PMC6337176; doi:10.3390/molecules24010198)
Supplement: Supplementary file 1 [file molecules-24-00198-s001.pdf]

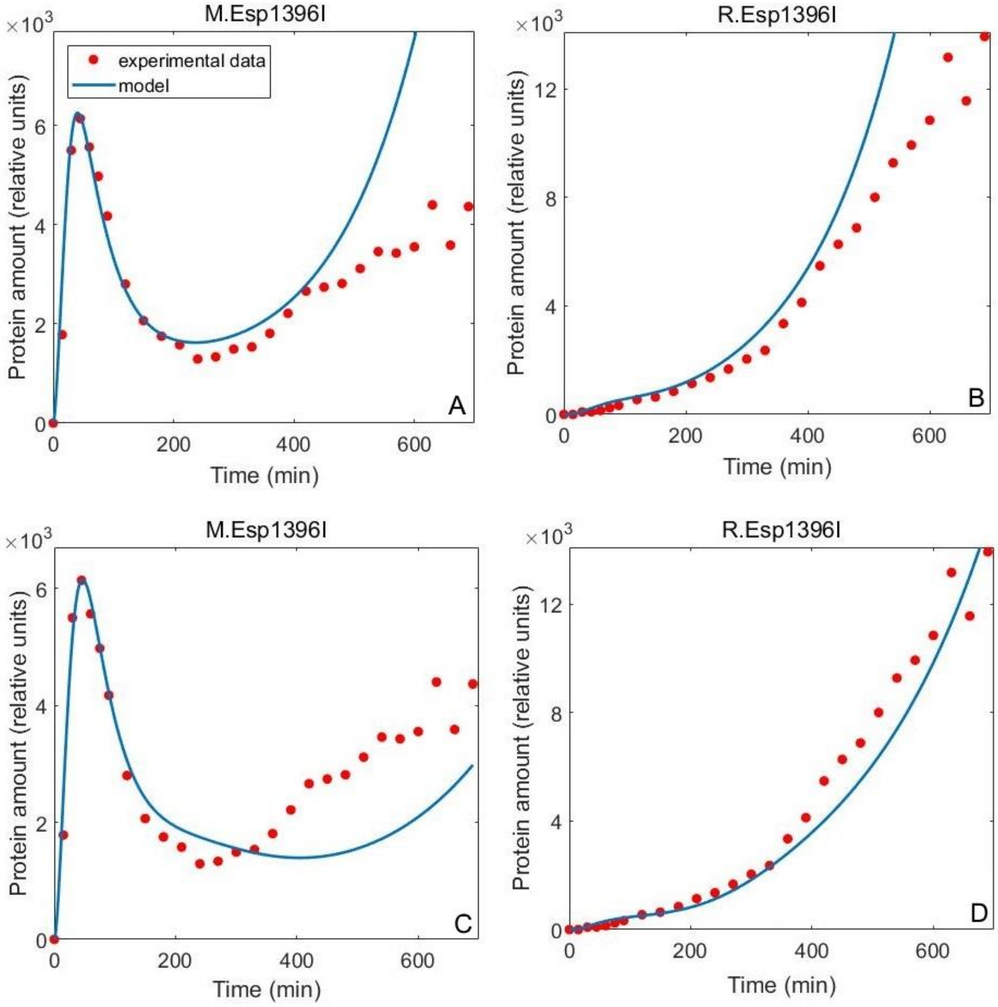

***F**igure S1 – Model with a single parameter describing plasmid division dynamics. (A)* The single parameter describing plasmid division dynamics is assigned the value of the first plasmid division rate from the three-parameter model. The model including this assumption is simulated (blue curve) and compared to the data (red dots) *(A)* for methyltransferase, where F value for the comparison with the fit in Fig. 3A is 938 (P~10^{-22}), and *(B)* restriction endonuclease dynamics, with F=1782 (P~10^{-25}) for comparison with the fit in Fig. 3B. *(C)* The model containing a single free parameter describing plasmid division dynamics is fitted to the experimental data for methyltransferase, with F=60 (P~10^{-9}), and *(D)* restriction endonuclease dynamics, with F = 10 (P~10^{-3}).

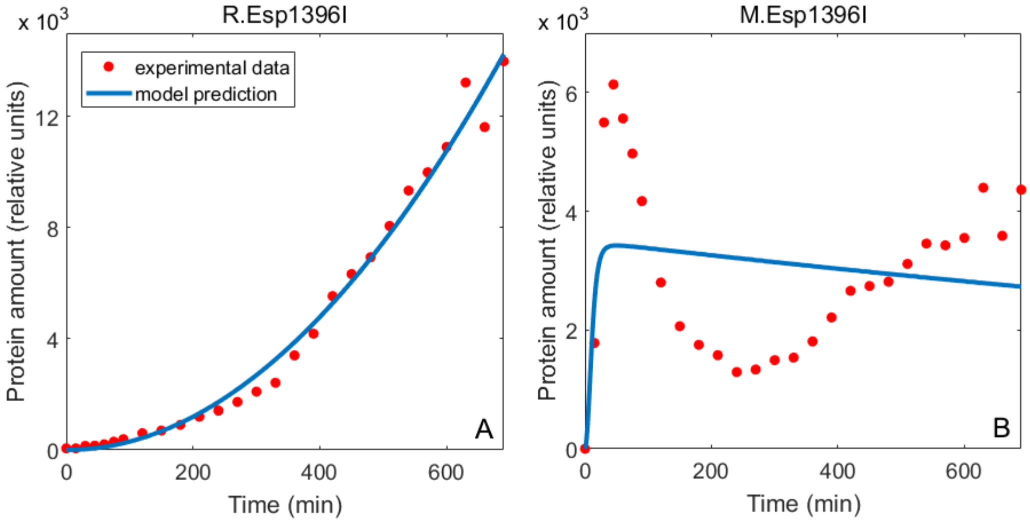

Figure S2 – Model ignoring population dynamics fitted to the experimental data for **(A)** restriction endonuclease and **(B)** methyltransferase expression dynamics.
